# Organic Solvent Nanofiltration of Water-in-Oil Pickering Emulsions—What Influences Permeability?

**DOI:** 10.3390/membranes11110864

**Published:** 2021-11-10

**Authors:** Maresa Vivien Kempin, Anja Drews

**Affiliations:** Department II, Chemical Engineering in Life Science Engineering, HTW Berlin—University of Applied Sciences, Wilhelminenhofstraße 75A, 12459 Berlin, Germany; anja.drews@htw-berlin.de

**Keywords:** crossflow velocity, dispersed phase fraction, emulsion composition, mathematical modeling, organic solvent nanofiltration, organic solvent type, particle concentration, particle type, Pickering emulsions

## Abstract

Pickering emulsions (PEs) have received increasing interest for their application in catalytic multiphase reactions. Organic solvent nanofiltration of PEs was shown to be a promising procedure for efficient and effective catalyst recycling. In this work, a systematic parameter study to identify the main influencing parameters on PE filtration was conducted for a large variety of PE compositions for the first time. In addition to temperature, only the type of organic solvent significantly influenced the filtration performance, which could be mathematically modeled via a combination of the solution–diffusion and the resistance in the series model. Particle type and concentration, dispersed phase fraction and the presence of reaction (by-)products did not show any significant impact on the permeability. The stirrer speed only became important when emulsions stabilized by particles without the tendency to form 3D network structures were filtered in long-term filtration experiments. These results pave the way towards the application of PE membrane filtration for catalyst recovery in continuous liquid/liquid multiphase reactions and enable broad operation windows. As the mechanical separation of PEs was shown to be a very robust process, the emulsion composition can now be tuned to meet the needs of the reaction without any (significant) loss in filtration performance.

## 1. Introduction

Pickering emulsions (PEs), named after S.U. Pickering [1], are stabilized by solid nanoparticles and are known for their superior stability [2]. Therefore, their application in (bio-)catalytic liquid/liquid (L/L) reactions has received increasing attention within the last decade, e.g., [3,4,5,6]. However, efficient and effective catalyst recovery to obtain economically feasible processes remained a challenge until recently [7]. Most publications used process concepts based on demulsification (either via centrifugation [4] or stimuli-responsiveness, e.g., [8,9]) which only allow cyclic processes and might destroy sensitive components (e.g., enzymes). In 2016, the feasibility of water-in-oil (w/o) PE ultrafiltration was shown for the first time [10]. The catalyst could be immobilized within the dispersed aqueous phase droplets, while the product containing organic phase was continuously filtered through the membrane. Since then, some studies on the ultrafiltration of w/o PEs were published [5,11,12,13,14]. However, the ultrafiltration membranes used in these studies (molecular weight cut-off (MWCO) ranging from 1 to 10 kDa) were originally designed for aqueous applications and showed—in some cases—an unexpected disproportionate increase of flux with pressure and strong fluctuations in pure organic solvent flux [10,11,13,14]. Therefore, an organic solvent nanofiltration membrane with a relatively high MWCO of 900 Da was tested for w/o PE filtration for the first time in [13,15]. Since the droplets are expected to be in the µm-range [14] and possibly freely suspended particle aggregates have a size of approximately 150 nm [16], membranes with a smaller MWCO were not necessarily needed. Despite the low applied pressures (1–4 bar, based on the first investigations using ultrafiltration membranes), in [13,15], feasible and very reproducible fluxes could be achieved using the organic liquid 1-dodecene (pure organic solvent flux: *J* (*p* = 4 bar) = 14.34 ± 2.38 Lm^−2^h^−1^). So far, the impact of drop size distribution (PEs of constant composition were prepared using different homogenization conditions of a batch rotor-stator device) [13] and temperature [15] on the w/o PE filtration behavior was investigated. An expected increase in flux with increasing Sauter mean diameters could not be clearly confirmed in the previous studies [12,13]. An increase in temperature led to an increase in flux and could be modeled via a combination of the solution–diffusion and the resistance in series model including an Arrhenius-type relationship to describe the temperature dependency of the diffusion coefficient [15].

However, a systematic parameter variation using an organic solvent nanofiltration membrane—including the PE composition and the operating conditions—has, to the best of our knowledge, not been conducted before. Therefore, the aim of this paper is to define the main influencing parameters on w/o PE filtration, based on such a systematic parameter study. The following parameters were varied:

- Particle type;
- Particle mass fraction;
- Dispersed phase fraction;
- Presence of catalyst and reaction (by-)products;
- Shear rate/crossflow velocity;
- Organic solvent type.

Furthermore, the validity of the proposed mathematical model to describe the filtration of w/o PEs developed in [15] was tested for different organic solvents and was adapted to include properties of the solvents.

## 2. Materials and Methods

### 2.1. Preparation of w/o Pickering Emulsions

Water-in-oil PEs of different compositions were investigated in this work, varying the particle type, the particle mass and the dispersed phase fraction, the presence of catalyst and reaction (by-)products as well as the organic solvent type. For all emulsions, deionized water was used as the aqueous phase. Based on our previous work [13,14,15,17,18], 1-dodecene was used as the “standard” organic solvent (VWR International GmbH, Dresden, Germany), as it constitutes a representative long-chain olefin suited for, e.g., hydroformylation reactions (e.g., [18]). Additionally, dodecane (Fisher Scientific GmbH, Schwerte, Germany), decene (Sigma-Aldrich Chemie GmbH, Taufkirchen, Germany), octene (Fisher Scientific GmbH, Schwerte, Germany) and heptane (Th. Geyer GmbH & Co. KG, Berlin, Germany) were investigated (Table 1). All chemicals were used as received.

**Table 1:** Characteristic properties of the organic solvents used in this study. Molar masses and densities were taken from [19]. Dynamic viscosities were measured as described in Section 2.3.

Component	CAS-Nr.	Purity	$\tilde{M}$	*ρ* (20 °C)	*η* (20 °C)
	[−]	[%]	[g mol^−1^]	[kg m^−3^]	[mPa s]
1-dodecene	112-41-4	for synthesis	168.32	758.4	1.25
dodecane	112-40-3	>99.0	170.33	749.5	1.44
decene	872-05-9	>97.0	140.27	740.8	0.71
octene	111-66-0	>99.0	112.21	714.9	0.29
heptane	142-82-5	>99.2	100.20	680.0	0.36

Different types of silica nanoparticles were used (Table 2). Commercially available fumed fractal-like particles of varying hydrophobicity and specific particle surface area by Wacker Chemie AG (as published in our previous works [14,15]) and spherical in-house particles differing in charge or size (as published in our previous work [18]) were investigated. The particles will be referred to by the short names given in Table 2 in the following.

**Table 2:** Overview of nanoparticle types used in this work. The particle hydrophobicity is expressed either via the residual silanol content (% SiOH) or the contact angle (°).

Short Name	Particle Type	Access to Particles	Particle Shape	Size	Hydrophobicity	Ref.
H15	HDK^®^H15	commercial	fractal-like	50–150 nm	50%	[20]
H20	HDK^®^H20				50%	[21]
H30	HDK^®^H30				50%	[22]
H2000	HDK^®^H2000				25%	[23]
50−	50 C18n−	in-house	spherical	50 nm	113°	[18]
50+	50 C18n+			50 nm	107°	
100+	100 C18n+			100 nm	105°	

Particle mass fractions of either 0.5 or 1.0 wt.% (with respect to the total mass of the emulsion) were used. Typically, a dispersed phase fraction of 0.25 *v*/*v* was used. Based on our previous work, particle types H20 and H2000—differing in particle hydrophobicity—were used and compared in most of the investigations shown here. All emulsions were prepared using a batch rotor–stator homogenizer (IKA^®^-Werke GmbH & Co. KG, Staufen, Germany; IKA T 25 digital ULTRA-TURRAX^®^, *P_max_* = 500 W). While PEs stabilized by particle types H15, H20, H30 and H2000 were prepared using the S25N-18G dispersing head at constant homogenization conditions of 17,500 min^−1^/2 min [13], PEs stabilized by particle types 50−, 50+ and 100+ were prepared using the S25N-10G dispersing head at 20,000 min^−1^/5 min [18]. All experiments were conducted with individually prepared emulsions at room temperature (*T* = 22.2 ± 1.3 °C).

### 2.2. Drop Size Distribution

Drop size distributions were investigated using optical microscopy (Carl Zeiss Microscopy GmbH, Göttingen, Germany; Axio Scope.A1, 20-fold magnification). At least 25 pictures or 500 drops per emulsion sample, respectively, were taken to obtain statistically sound results. To minimize overlapping and clustering of droplets, PEs were diluted with the pure continuous phase. This was shown not to change the drop size distribution in [24]. The microscopic images were then analyzed using an image analysis software (SOPAT GmbH) [25]. Sauter mean diameters were calculated from number-based drop size distributions; compare Equation (1) (*n*—number of droplets).

(1) $$d_{32}=\sum_{i=1}^{n}d_{P,i}^{3}/\sum_{i=1}^{n}d_{P,i}^{2}.$$

### 2.3. Rheological Behavior

A cone and plate rheometer (Anton Paar Germany GmbH, Ostfildern, Germany; MCR 302, measurement system CP50-1: cone diameter 49.969 mm, angle 0.997°, gap size 0.102 µm) was used to investigate the rheological behavior of the pure organic solvents. All measurements were conducted at *T* = 20.0 ± 0.1 °C. The rheological measurement parameters were adapted from [26] and are consistent with the ones in our previous studies [13,14,17]. The sample was first sheared at a constant shear rate of 400 s^−1^ for 5 min, followed by an increase of shear rate from 1–1000 s^−1^.

### 2.4. Permeation of Pure Solvents and Filtration of w/o Pickering Emulsions

All filtration experiments were conducted in a solvent resistant stirred cell (XFUF04701 by Merck KGaA, Darmstadt, Germany; working volume: 91.5 mL, effective membrane area: 13.2 cm^2^). Consistent with our previous work [13], the organic solvent nanofiltration membrane oNF-3 of silicone polymer-based composite type was used (BORSIG Membrane Technology GmbH, Gladbeck, Germany; MWCO = 900 Da [27]). Due to this relatively high MWCO, relevant pure organic solvent fluxes could be achieved even at the lower pressures applied in this work. A new membrane sample was used for each experiment and LABVIEW was used to read an electronic balance and to record permeate mass over time. A detailed description of the filtration set-up and procedures can be found in, e.g., [11]. If not stated otherwise, a stirrer speed of 500 min^−1^ was applied. Consistent with our previous work (e.g., [12,15]), the retention of dispersed phase droplets and potentially freely suspended nanoparticles was 100%.

#### 2.4.1. Pre-Treatment of the Membrane

Membrane samples were soaked in the pure organic phase at least one day prior to use and were washed with the organic phase for 90 min at a constant pressure of 4 bar to achieve complete wetting, swelling and pre-compaction of the membrane prior to the actual experiment. A new membrane sample was used for each experiment.

#### 2.4.2. Pressure Stepping Experiments

Filtration experiments were typically conducted in pressure stepping mode at a constant dispersed phase fraction within the stirred cell as the organic phase was continuously transported from a feed tank to the stirred cell at the same rate as permeate was extracted when pressure was applied. The pressure was first increased in steps of 1 bar (from 1 to 4 bar) and then decreased again. Each pressure step was kept constant for half an hour and steady state fluxes were always reached. Permeabilities were calculated for each pressure step and the average as well as standard deviations are shown.

#### 2.4.3. Long-Term Filtration Experiments

Some selected experiments were conducted at a constant pressure of 4 bar for 5 h at constant dispersed phase fraction within the stirred cell.

#### 2.4.4. Concentration Experiments

Some selected concentrating experiments were conducted at a constant pressure of 4 bar. Therefore, feed was not supplied but the dispersed phase fraction within the filtration cell increased with time and was calculated using a mass balance (as described in more detail in [11]).

## 3. Results

### 3.1. Impact of Particle Type

**Figure 1:**
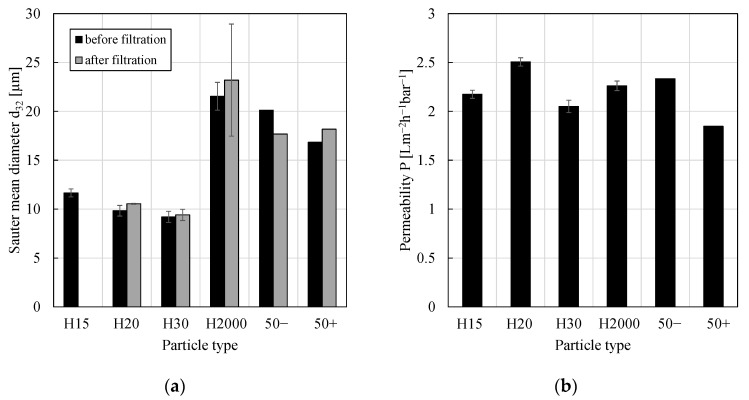
(**a**) Sauter mean diameter and (**b**) permeability of w/o PEs stabilized by 0.5 wt.% of different silica particles. The PEs stabilized by particle types 50− and 50+ were investigated once and used for hydroformylation reactions (and subsequent concentration filtration experiments). All other emulsions were prepared without the catalyst-ligand complex and investigated at least in duplicate pressure stepping experiments. Mean values are shown and error bars represent the standard deviation. Drop size distributions for particle types H15, H20, H30 and H2000 were already published in [14] and Sauter mean diameters for particle types 50− and 50+ were published in [18].

The w/o PEs stabilized by 0.5 wt.% of the different nanoparticle types, listed in Table 2, were investigated. The Sauter mean diameters are given in Figure 1a. Consistent with results published in the literature [5,26,28], more hydrophobic silica particles (in this case, e.g., H2000 in comparison to particle types H15, H20 and H30) led to larger drop sizes and PEs stabilized by fumed silica particles (e.g., particle types H15, H20 and H30) showed smaller Sauter mean diameters than those stabilized by spherical ones (particle types 50− and 50+). Except for emulsions stabilized by H15 particles, the PEs were stable against the applied shear and pressure during the filtration experiments since—within the experimental error—no change in the average drop size could be observed. Experimental data concerning the rheological behavior of PEs stabilized by particle types H15, H20, H30 and H2000 were already published in [14] with emulsions stabilized by particle types H15, H20 and H30 showing shear-thinning rheological behavior (and the ability to form three-dimensional network structures) while emulsions stabilized by particle type H2000 showed a Newtonian flow behavior (and no ability to form networks).

Permeabilities (Equation (2)) of PEs stabilized by different particle types are given in Figure 1b. Particle types H15, H20, H30 and H2000 were investigated in pressure stepping experiments (permeability was calculated for each pressure step and mean values are shown; flux was constant during each pressure step) while particle types 50− and 50+ were investigated in concentration experiments (flux was constant with time; concentration experiments were stopped when half of the continuous phase was obtained as permeate).

(2) $$P=\frac{J}{\Delta p}.$$

Within (or at least close to) the experimental error, permeabilities were similar and no clear tendency or significant effect of particle hydrophobicity, particle shape or drop sizes on the filtration behavior could be found. Furthermore, the presence of the catalyst–ligand complex (Rh-(acac)(CO)_2_ and sulfoxantphos) and (by-)products (tridecanal) from the hydroformylation reaction did not significantly change the permeability (PEs stabilized by particle types 50− and 50+, respectively; for details the reader is referred to [18]). For comparison, the permeability of pure 1-dodecene was 3.58 ± 0.60 Lm^−2^h^−1^bar^−1^. Consequently, all PEs reduced this permeability by only approximately 40%. Using the oNF-3 membrane, the particle type used for PE stabilization can be adjusted to optimize the actual reaction without any significant loss in filtration performance (e.g., small drop sizes for large interfacial area or specific particle charge to optimize particle–catalyst interactions [18]).

Contrary to the results shown here, using ultrafiltration membranes in [5,14], smaller fluxes or permeabilities, respectively, for particles without gelling-properties (without the ability to form three-dimensional network structures) in comparison to gelling particles were observed. In [14], particle types H15, H20, H30 and H2000 were investigated using the ultrafiltration membrane ETNA01PP. Contact angle measurements as well as the filtration of nanoparticle/oil suspensions revealed an increase in membrane wettability after silica particle contact. Comparison of the results from suspension filtrations using the ETNA01PP ultrafiltration membrane [14] and the oNF-3 organic solvent nanofiltration membrane (data not shown) showed that the impact of possibly freely suspended nanoparticles is much less pronounced in case of the oNF-3 membrane. Therefore, we assume that the different results regarding the impact of particle type on the filtration performance here and in [5,14] can be explained by different interactions between the particles and the membrane material and a possible change in membrane wettability.

### 3.2. Impact of Particle Concentration

**Figure 2:**
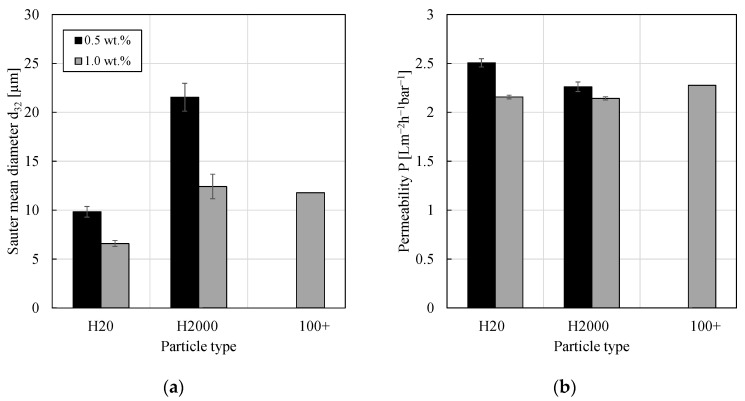
(**a**) Sauter mean diameter and (**b**) permeability of w/o PEs stabilized by either 0.5 or 1.0 wt.% of different silica particles. The PE stabilized by particle type 100+ was investigated once and used for a hydroformylation reaction (with subsequent concentration filtration experiment). All other emulsions were prepared without the catalyst-ligand complex and investigated at least in duplicate pressure stepping experiments. Mean values are shown and error bars represent the standard deviation. For comparison, the permeability of pure 1-dodecene was 3.58 ± 0.60 Lm^−2^h^−1^bar^−1^. Drop size distributions for particle types H20 and H2000 were already published in [14] and the Sauter mean diameter for particle type 100+ was published in [18].

Figure 2 shows the results of Sauter mean diameters and permeabilities for PEs stabilized by different particle mass fractions of different silica nanoparticle types. Consistent with the results from the literature, an increase in particle mass fraction led to a decrease in the average drop size as a greater interfacial area could be stabilized, e.g., [10,29,30]. Considering the change in *d*_32_ of approximately 33% or 42% (H20 or H2000, respectively), one would have expected a decrease of flux of 55% to 66% (flux proportional to the Sauter mean diameter squared using the Carman–Kozeny equation and assuming all other parameters in this equation as being constant). However, the impact on the permeability was low, compare Figure 2b. From Figure 2, one can—again—conclude that the presence of the catalyst-ligand complex and reaction (by-)products did not have a significant impact on the filtration performance (PE stabilized by particle type 100+).

Heyse et al. observed a decrease in permeability by approximately 40% when the particle concentration of in-house spherical, non-gelling silica particles was quadrupled (polyethersulfone (PES) membrane with a MWCO of 10 kDa, cyclopentyl methyl ether (CPME) as continuous phase) [12]. However, due to the different material pairings and different operating conditions during the filtration experiments, a direct comparison with our work is not possible. At high particle mass fractions, a further increase in the silica content no longer leads to a reduction of the droplet size but to an increase in the amount of excess particles in the continuous phase [30].

Thus, again, using the oNF-3 membrane, the composition of the PE can be optimized to meet the needs of the actual reaction without any significant loss in filtration performance. However, possible interactions between the nanoparticles, the solvent and the membrane need to be investigated. Furthermore, it must be considered that the adsorption of nanoparticles to the L/L interface on the one hand side leads to a reduction of the average drop size due to coalescence hindrance, while on the other side, the nanoparticle coverage reduces the interfacial area available for mass transfer to occur. Consequently, the additional mass transfer resistance at high nanoparticle concentrations has to be taken into account [31].

### 3.3. Impact of Dispersed Phase Fraction

**Figure 3:**
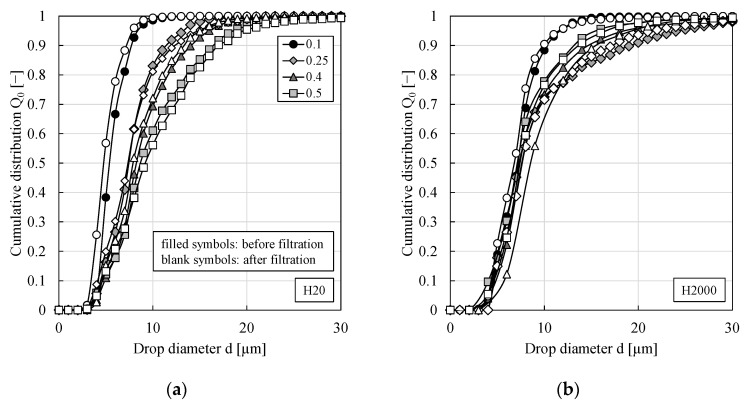
Cumulative distribution for w/o PEs stabilized by 0.5 wt.% of particle type (**a**) H20 or (**b**) H2000 and different dispersed phase fractions before and after the filtration. All experiments were performed at least in duplicate and mean values are shown. For better graph clarity, error bars are not shown but were always below 25%.

For PEs stabilized by 0.5 wt.% of particle type H20 or H2000, the impact of dispersed phase fraction on the drop size distribution was investigated, see Figure 3. For particle type H20, a continuous shift of the drop size distribution towards larger drop diameters was observed when the dispersed phase fraction was increased while for particle type H2000 no clear trend could be observed. In the literature, an increase of Sauter mean diameters upon an increase of the dispersed phase fraction has frequently been reported, e.g., [26,32,33,34,35]. Changing the water/oil ratio has an impact on the sample density and viscosity and consequently on the coalescence and breakup effects during emulsification [26]. As can be seen from Figure 3, all emulsions remained stable towards coalescence throughout the pressure stepping experiments as—within the experimental error—no significant shift of the drop size distributions could be observed.

**Figure 4:**
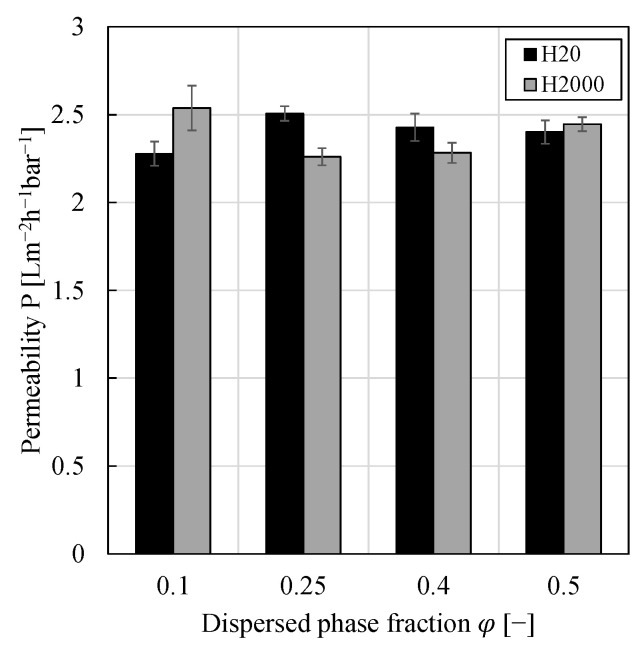
Permeability against dispersed phase fraction for w/o PEs stabilized by 0.5 wt.% of either particle type H20 or H2000. All pressure stepping experiments were conducted at least in duplicate and mean values are shown. Error bars represent the standard deviation. For comparison, the permeability of pure 1-dodecene was 3.58 ± 0.60 Lm^−2^h^−1^bar^−1^.

Figure 4 shows the impact of the dispersed phase fraction on the permeability. Even though the error bars do not overlap in all cases, considering the inherent differences of membrane samples, no significant impact on the filtration behavior could be observed. In our previous work [13], the impact of dispersed phase fraction was investigated by preparation of different volumes of PEs of otherwise constant composition using particle type H20 and subsequent dilution of the PEs in the stirred cell in order to obtain a completely filled cell (particle mass per liter of dispersed phase of 16.5 $gL_{dP}^{-1}$ for all PEs). In this work, equal volumes of PEs of varying compositions were prepared without any further dilution in the stirred cell (particle mass per liter of dispersed phase varied between approximately 9–40 $gL_{dP}^{-1}$). In both cases, Figure 4 and [13], the impact of the dispersed phase fraction was negligible allowing the filtration of highly concentrated emulsions, which can be beneficial for process intensification and allows small reactors.

### 3.4. Impact of Shear Rate/Crossflow Velocity

**Figure 5:**
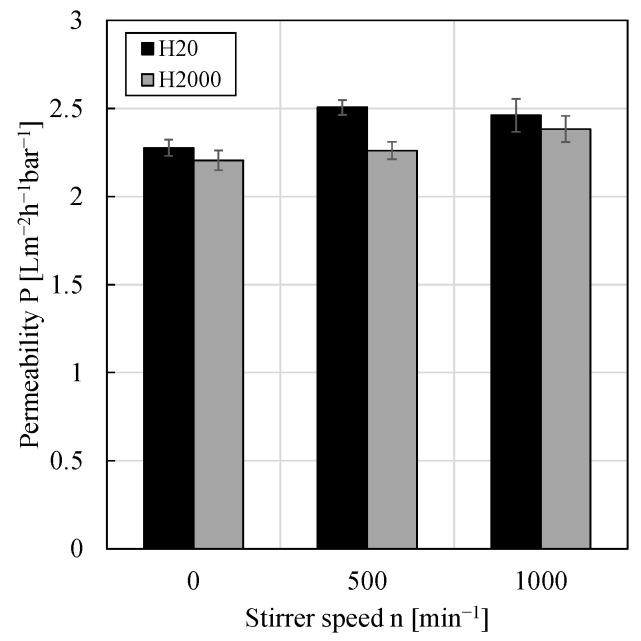
Permeability against stirrer speed within the stirred cell for w/o PEs stabilized by 0.5 wt.% of either particle type H20 or H2000. All pressure stepping experiments were conducted at least in duplicate and mean values are shown. Error bars represent the standard deviation. For comparison, the permeability of pure 1-dodecene was 3.58 ± 0.60 Lm^−2^h^−1^bar^−1^.

To the best of our knowledge, the impact of different shear rates or crossflow velocities, respectively, on the filtration of w/o PEs has never been investigated before. However, one could assume that compared to an unstirred system, stirring leads to a removal of droplets from the membrane surface and thus a smaller filter cake. Furthermore, applying different stirrer speeds or using PEs with different drop size distributions, one could assume droplet classification to occur as the crossflow creates a lift force (while the permeate creates a drag force) mainly lifting large droplets and keeping the smaller droplets in the deposit leading to a higher cake resistance. Within the used filtration cell, different stirrer speeds of the hanging stir bar could be applied. Thus, without stirring the filtration can be regarded as a dead-end filtration with cake heights constantly increasing over time while with stirring, it can be regarded as a mixed form since the stirring leads to a crossflow leading to a presumably constant filter cake height. Permeabilities calculated from pressure stepping experiments using the particle types H20 and H2000, respectively, as a function of the stirrer speed are shown in Figure 5. Contrary to the expectations, again, within the experimental error, no significant impact of the applied stirrer speed or shear rate, respectively, could be observed for either particle type.

**Figure 6:**
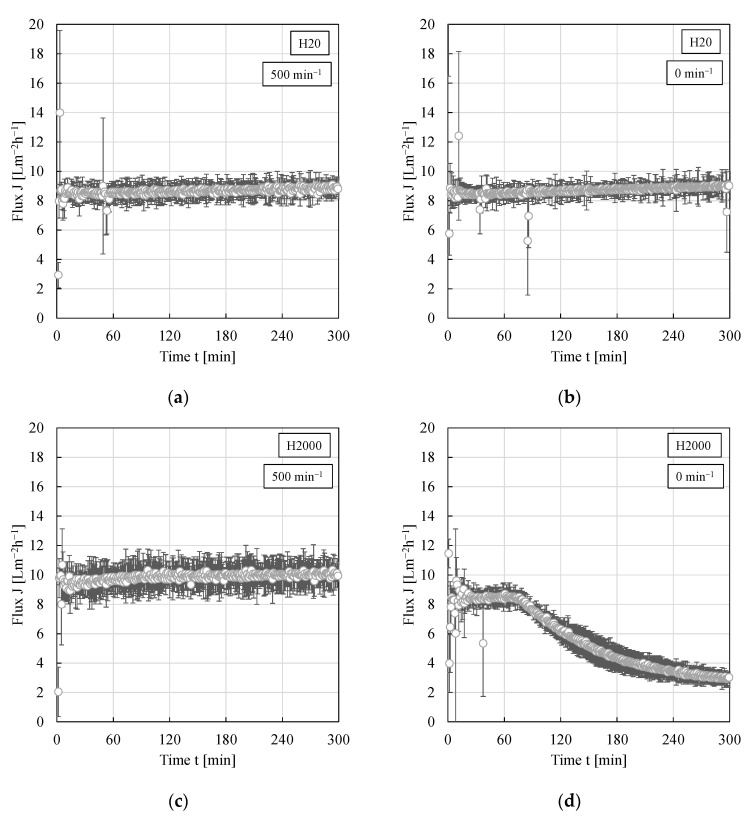
Flux as a function of time for w/o PEs stabilized by 0.5 wt.% of particle type (**a**,**b**) H20 or (**c**,**d**) H2000. Filtration experiments were conducted at a constant pressure of 4 bar and a constant dispersed phase fraction of 0.25 within the filtration cell. Experiments were conducted at least in duplicate and mean values are shown. Error bars represent the standard deviation.

However, during long-term filtration experiments (5 h at a constant pressure of 4 bar), the situation changed for particle type H2000 and an impact of the stirrer speed became apparent after approximately 75 min (compare Figure 6c,d). For the network forming particle type H20 [13,14], on the other hand, the flux remained constant over the entire period of time (compare Figure 6a,b). The very slight increase of flux with time can be traced back to an increase of lab temperature throughout the day (~2 °C; in our previous work a 5 °C increase in temperature resulted in an increase in PE flux of approximately 1.6 Lm^−2^h^−1^ [15]).

The decrease of flux for PEs stabilized by particle type H2000 without stirring can probably be traced back to the different sedimentation velocities of the µm-sized emulsion droplets compared to the nm-sized particles. For a first estimation, the drop or particle sedimentation velocities were calculated (assumption of spherical (individual) drops or particles, without and with superposition of the flux). For a 20 µm big droplet it takes less than 5 min to settle over the entire height of the filtration cell to the membrane surface (the superposition of the flux does not significantly reduce this time). For a nm-sized particle (approximately 150 nm [16]); however, the superposition of the flux significantly reduces the sedimentation time. After 75 min, all particles that were in an approximately 1.3 cm thick layer above the membrane have settled to the membrane surface. Consequently, at the beginning of the experiment, the filter cake mainly constitutes of emulsion droplets while with time, more nanoparticle (aggregates) can settle in the voids between the emulsion droplets. As known from nanoparticle/oil suspension filtrations without stirring [14], a dense filter cake increasing the cake resistance is then formed when particle type H2000 is used.

### 3.5. Impact of Organic Solvent Type

#### 3.5.1. Pure Organic Solvent Flux

In a first step, the permeation of the pure solvents through the oNF-3 membrane was investigated in pressure stepping experiments and was mathematically modeled. Based on our previous work [15], the solution–diffusion model, comparing Equation (3), was used.

(3) $$J_{i}=\frac{D_{iM}}{\delta_{0}}\;\frac{c_{iM}\;{\tilde{V}}_{i}\;{\tilde{M}}_{i}}{ℜ\;T\;\rho_{i}\;S}\;\Delta p.$$

Herein, the subscript i denotes the organic solvent and *M* denotes the membrane; *J* is the flux, *D* is the diffusion coefficient, *c* is the concentration of solvent inside the membrane, $\tilde{V}$ is the molar volume, $\tilde{M}\;$is the molar mass, $\delta_{0}$ is the thickness of the dry active membrane layer, ℜ is the universal gas constant, *T* is the temperature, *ρ* is the density, *S* is the swelling degree and Δp is the transmembrane pressure difference. The operating conditions (*T*, *p*) were already defined in Section 2, the characteristic properties of the different organic solvents were given in Table 1, leaving the diffusion coefficient, the thickness of the dry active membrane layer, the concentration of solvent inside the membrane and the swelling degree as unknown parameters which had to be identified first. As described and found suited in our previous work [15], the parameter determination was based on [36], where the retention of surfactants from 1-dodecene via the organic solvent nanofiltration membranes oNF-1 and oNF-2 was investigated. The concentration of solvent inside the membrane was calculated using Equation (4) with 1 g of polydimethylsiloxane (PDMS) having a volume of $V_{PDMS}$ = 1.03 cm^3^ and $V_{fV}$ = 0.2 cm^3^ taken from [36] and kept constant for all organic solvents used.

(4) $$c_{iM}=\frac{n_{i}}{V_{total}}=\frac{m_{i}/{\tilde{M}}_{i}}{V_{PDMS}-V_{fV}+V_{solvent}}.$$

The mass of solvent inside the membrane was experimentally detected via gravimetric absorption measurements at room temperature and is given in Table 3 together with the calculated concentration of solvent inside the membrane. In this work, only the composite membrane was available, while Zedel et al. [36] conducted their experiments with a pure PDMS layer. However, the experimentally determined values for 1-dodecene are in great agreement with the values published in [36] ($m_{i}$ = 1.6 g and $c_{iM}$ = 3240 mol m^−3^).

The swelling degree *S* was experimentally determined via thickness measurements of a dry and swollen PDMS layer in [36] and found to be 2 for 1-dodecene. As mentioned, in this work only the composite membrane was available and consequently measurements of the swelling degree for the different solvents were not possible. Therefore, for this first modeling approach, a constant value of *S* = 2 was assumed.

**Figure 7:**
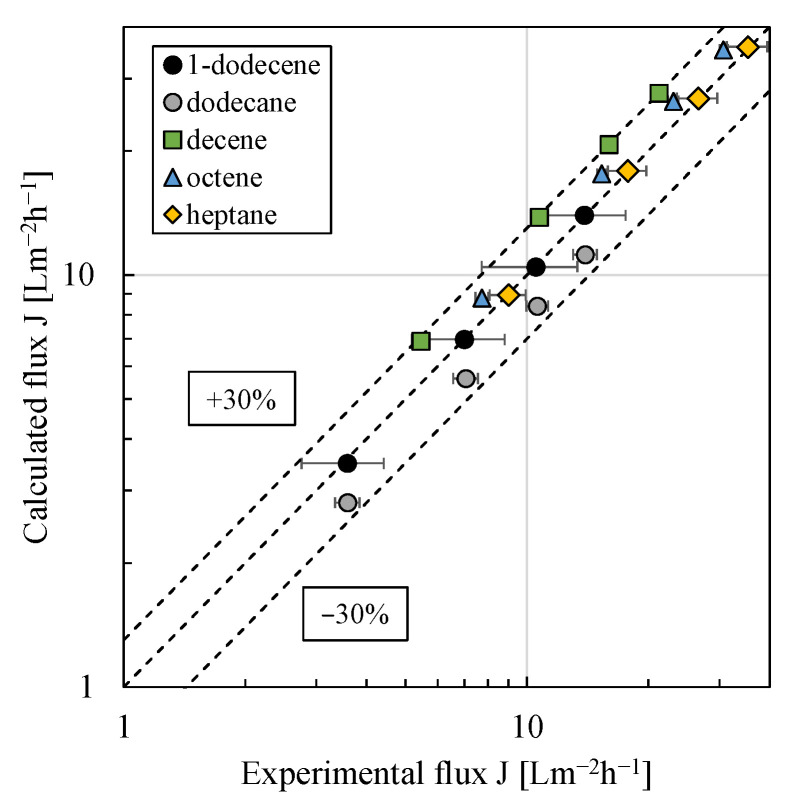
Calculated vs. experimental flux using the solution–diffusion model and a linear correlation between the ratio of (diffusion coefficient/dry active membrane layer thickness) and the molar volume of the organic solvent (Equations (3) and (5)). For the model fit, the experimental data of 1-dodecene and heptane were used. All pressure stepping experiments were conducted at least in duplicate and mean values are shown. Error bars represent the standard deviation. Where not visible, error bars are smaller than the symbol size.

In our previous work [13,15], it was shown that the oNF-3 membrane is incompressible within the experimentally investigated pressure range (1–4 bar). The unknown but constant dry active layer thickness of the oNF-3 membrane was therefore lumped with the diffusion coefficient in the fitting. In [15], the temperature dependency of the diffusion coefficient could be described via an Arrhenius-type relationship. In [37], the diffusion coefficient was found to depend inversely on the molecular size of the penetrating molecule and a linear correlation between the diffusion coefficient and the molar volume of the organic solvent was proposed. However, using the experimental data for 1-dodecene and heptane for such a linear correlation, compare Equation (5), the differences between experimental and modeled flux values were relatively high (up to 30%) as can be seen in the parity plot in Figure 7. A systematic deviation was observed as calculated fluxes for decene were too high and those for dodecane were too small (regardless of the pressure).

(5) $$\frac{D_{iM}}{\delta_{0}}=-1.23\;10^{-5}\;{\tilde{V}}_{i\;}\frac{mol}{m^{2}s}+0.003\;\frac{m}{s}.$$

**Figure 8:**
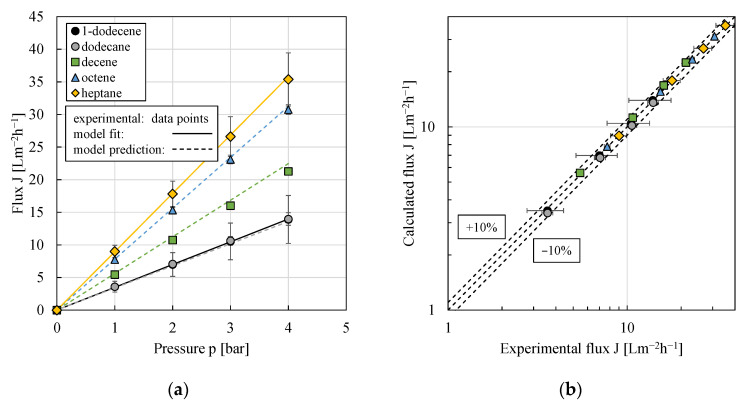
(**a**) Flux against pressure (for better graph clarity, only the results for decreasing pressures are shown) and (**b**) calculated flux vs. experimental flux using the solution–diffusion model and a linear correlation between the ratio of (diffusion coefficient/dry active membrane layer thickness) and the reciprocal of the molar mass of the organic solvent (Equations (3) and (6)). Experimental data points for 1-dodecene and dodecane overlap. For the model fit, the experimental data of 1-dodecene and heptane were used. All pressure stepping experiments were conducted at least in duplicate and mean values are shown. Error bars represent the standard deviation. Where not visible, error bars are smaller than the symbol size.

A significantly better model prediction was obtained, when the ratio of diffusion coefficient and dry active membrane layer thickness was linearly correlated with the reciprocal of the molar mass of the organic solvent as another measure of the molecular size; compare Equation (6) and Figure 8. Consequently, when correlating via the molar mass, the exact knowledge of the swelling degree for each solvent is not necessary and the assumption made (S = 2 for all solvents) is sufficient.

(6) $$\frac{D_{iM}}{\delta_{0}}=\left(0.2272\;\frac{1}{{\tilde{M}}_{i}}\;\frac{g}{mol}-0.001\right)\;\frac{m}{s}.$$

Deviations between experimental and modeled flux values were smaller than 10% (Figure 8b). Therefore, this mathematical modeling approach will be used in the following.

#### 3.5.2. Filtration of w/o PEs Using Different Organic Solvents

**Figure 9:**
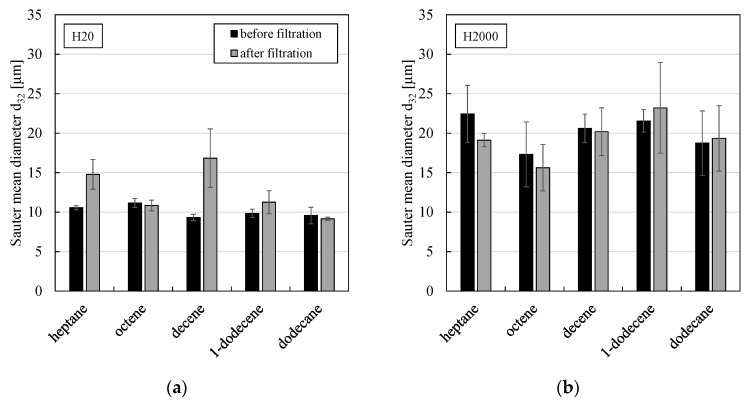
Sauter mean diameter against organic solvent type of w/o PEs stabilized by 0.5 wt.% of particle type (**a**) H20 or (**b**) H2000 before and after the filtration. All experiments were conducted at least in duplicate and mean values are shown. Error bars represent the standard deviation.

Figure 9 shows Sauter mean diameters of w/o PEs stabilized by either particle type H20 or H2000, respectively, and prepared using different organic solvents before and after the filtration. For all solvents, the more hydrophobic particle type H2000 led to larger Sauter mean diameters, a greater polydispersity and emulsion preparation was not as reproducible compared to particle type H20 (indicated by larger error bars). Except for H20 stabilized heptane and decene emulsions, most emulsions remained stable towards coalescence throughout the filtration as the Sauter mean diameter did not change significantly (within the experimental error).

Based on our previous work [15], a combination of the solution–diffusion model and a resistance in the series model to describe the filtration behavior of PEs, compare Equation (7), was used.

(7) $$\frac{1}{J_{PE,k}}=\frac{\delta_{0}\;ℜ\;T\;\rho_{i}\;S}{D_{iM}\;c_{iM}\;{\tilde{V}}_{i}\;{\tilde{M}}_{i}\;\Delta p}+\frac{\eta_{i}\;R_{C,k}}{\Delta p}.$$

**Figure 10:**
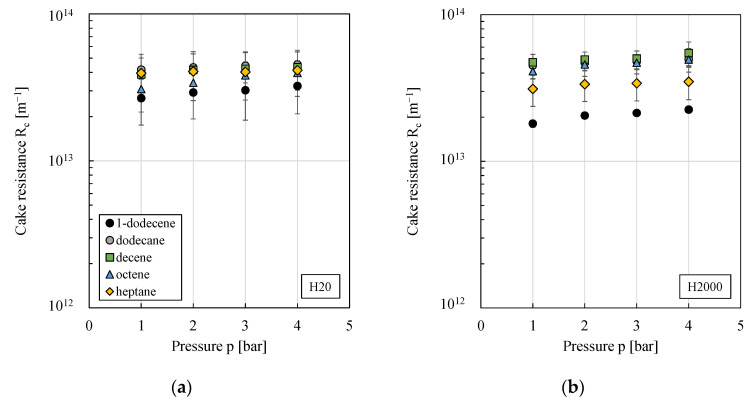
Cake resistance against pressure for w/o PEs prepared using different organic solvents and stabilized by particle type (**a**) H20 or (**b**) H2000. All pressure stepping experiments were conducted at least in duplicate and mean values are shown. Error bars represent the standard deviation. Where not visible, error bars are smaller than the symbol size.

In this equation, only the cake resistance $R_{C}$ (the subscript k denotes the particle type) is unknown. Therefore, using the resistance in the series model, cake resistances were calculated from experimental data with the membrane resistance calculated via Darcy’s law using the results from the permeation of the pure organic solvents ($R_{M}$ was in the range of 7.1·10^13^–1.6·10^14^ m^−1^; data not shown). As can be seen from Figure 10, cake resistances were in a similar range for both particle types and—within the experimental error—independent of pressure. Consequently, for the investigated pressure range, cake resistances were assumed to be incompressible. Scatter of cake resistances for the different organic solvents was higher. However, no clear correlation of cake resistances with, e.g., the solvents characteristics or the Sauter mean diameters (compare Figure 9) could be found.

**Figure 11:**
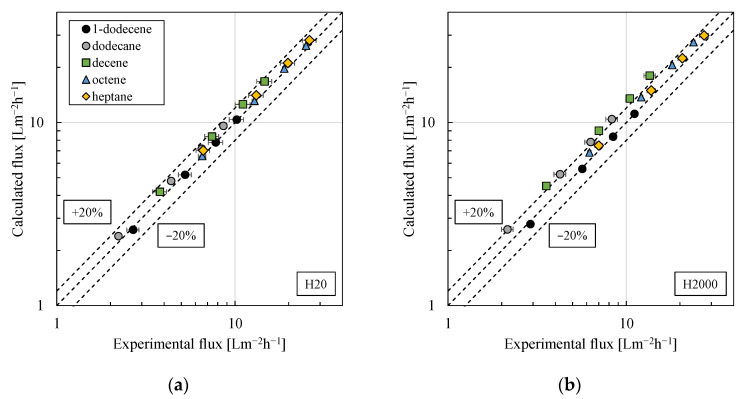
Calculated flux vs. experimental flux for w/o PEs prepared using different organic solvents and stabilized by particle type (**a**) H20 or (**b**) H2000. For the model fit (solution–diffusion model combined with a resistance in series model, compare Equation (7)), the experimental results of 1-dodecene were used. All pressure stepping experiments were conducted at least in duplicate and mean values are shown. Error bars represent the standard deviation. Where not visible, error bars are smaller than the symbol size.

Therefore, a constant value—obtained from pressure stepping experiments of 1-dodecene at room temperature—was used for the model fit in this first mathematical modeling approach (3.07·10^13^ m^−1^ for particle type H20 and 2.23·10^13^ m^−1^ for particle type H2000). Implementing this into Equation (7) (combined with Equation (6)), yielded deviations between experimental and calculated fluxes of approximately 20%, compare Figure 11. Considering the assumptions made, the results can be rated as good. Furthermore, the model is of great practical applicability, since only a very limited amount of (filtration) experiments was needed for the model fit.

## 4. Conclusions

In this work, the impact of PE composition as well as operating conditions on the filtration behavior of w/o PEs using the organic solvent nanofiltration membrane oNF-3 was systematically investigated for the first time. Interestingly, among the PE compositions, only the organic solvent type (dynamic viscosity and diffusion coefficient, respectively) had a significant effect on the filtration behavior. The nanoparticle type and concentration, the dispersed phase fraction or the presence of catalyst–ligand complexes and reaction (by-)products, however, did not significantly affect the permeabilities obtained from filtration experiments. Regarding the process conditions, the pressure and the temperature [15] were identified as the main influencing parameters. The stirrer speed applied within the filtration cell only became important when PEs stabilized by particles without the tendency to form three-dimensional network structures between emulsion droplets and/or nanoparticles were filtered in long-term filtration experiments.

The mathematical model to describe the filtration of w/o PEs at different temperatures—developed in [15]—could successfully be adopted to describe the filtration performance of PEs prepared using different organic solvents. A combination of the solution–diffusion model with a resistance in the series model yielded a good agreement between experimental and calculated fluxes (deviations smaller than 10% for pure solvents; deviations smaller than 20% for PEs). The diffusion coefficient was linearly correlated with the reciprocal of the molar mass of the solvent and cake resistances could be assumed to be independent of pressure and solvent type. The developed model is of great practical applicability, since only a very limited number of experiments were required for the model fit.

The results from this work show that the mechanical separation of PEs via membrane filtration (using the organic solvent nanofiltration membrane oNF-3) is a very robust process and is thus well suited for catalyst recovery in continuous catalytic L/L multiphase reactions. As the filtration behavior was only affected by a limited number of parameters, broad operation windows are possible and PEs can be optimized for the actual reaction step without compromising the feasibility of PE membrane filtration. These results—together with the mathematical model to describe the filtration behavior of PEs—are indispensable for a model-based optimal process design for PE application in continuous catalytic L/L multiphase systems.

## Figures and Tables

**Table 3:** Mass and concentration of organic solvent inside the oNF-3 membrane samples. Gravimetric absorption experiments were conducted with five individual membrane samples. Mean values and standard deviations are given.

Organic Solvent	*m_i_*	*c_im_*
	[g]	[mol m^−3^]
1-dodecene	1.58 ± 0.03	3214.8
dodecane	1.57 ± 0.03	3148.3
decene	1.51 ± 0.02	3746.5
octene	1.36 ± 0.03	4429.5
heptane	1.20 ± 0.03	4613.5

## Data Availability

The data is included in the paper (in the form of diagrams). For exact values, please contact the corresponding author.
